# *AP2/ERF* genes associated with superfast fig (*Ficus carica* L.) fruit ripening

**DOI:** 10.3389/fpls.2022.1040796

**Published:** 2022-10-31

**Authors:** Yuanyuan Cui, Yanlei Zhai, Jiajun He, Miaoyu Song, Moshe A. Flaishman, Huiqin Ma

**Affiliations:** ^1^ Department of Fruit Tree Sciences, College of Horticulture, China Agricultural University, Beijing, China; ^2^ Peking University Institute of Advanced Agricultural Science, Shandong Laboratory for Advanced Agricultural Sciences, Weifang, China; ^3^ Department of Fruit Tree Sciences, Agricultural Research Organization, The Volcani Center, Bet Dagan, Israel

**Keywords:** ethylene response factors, expression pattern, gene structure, genome-wide identification, fruit development, transcriptome

## Abstract

Fig fruits have significant health value and are culturally important. Under suitable climatic conditions, fig fruits undergo a superfast ripening process, nearly doubling in size, weight, and sugar content over three days in parallel with a sharp decrease in firmness. In this study, 119 *FcAP2/ERF* genes were identified in the fig genome, namely 95 *ERF*s, 20 *AP2*s, three *RAV*s, and one *soloist*. Most of the ERF subfamily members (76) contained no introns, whereas the majority of the AP2 subfamily members had at least two introns each. Three previously published transcriptome datasets were mined to discover expression patterns, encompassing the fruit peel and flesh of the ‘Purple Peel’ cultivar at six developmental stages; the fruit receptacle and flesh of the ‘Brown Turkey’ cultivar after ethephon treatment; and the receptacle and flesh of parthenocarpic and pollinated fruits of the ‘Brown Turkey’ cultivar. Eighty-three *FcAP2/ERF*s (68 *ERF*s, 13 *AP2*s, one *RAV*, and one *soloist*) were expressed in the combined transcriptome dataset. Most *FcAP2/ERF*s were significantly downregulated (|log_2_(fold change) | ≥ 1 and *p*-adjust < 0.05) during both normal fruit development and ethephon-induced accelerated ripening, suggesting a repressive role of these genes in fruit ripening. Five significantly downregulated ERFs also had repression domains in the C-terminal. Seven *FcAP2/ERF*s were identified as differentially expressed during ripening in all three transcriptome datasets. These genes were strong candidates for future functional genetic studies to elucidate the major FcAP2/ERF regulators of the superfast fig fruit ripening process.

## Introduction

Transcription factors play important roles in plant signal transduction by activating or repressing the expression of target genes (Liu and Stewart, 2016). The APETALA2/ETHYLENE RESPONSIVE FACTOR (AP2/ERF) superfamily is a large class of transcription factors that are unique to plants (Licausi et al., 2013). All members of the AP2/ERF superfamily share a conserved AP2 domain, which has an amino acid (aa) length of approximately 60-70 and consists of a three-stranded β-sheet and one α-helix (Allen et al., 1998). AP2/ERFs can be divided into three subfamilies (ERF, AP2, and RAV) based on the number and aa sequences of AP2 domains present (Sakuma et al., 2002; Nakano et al., 2006). The ERF subfamily is the largest and is characterized by the presence of only one AP2 domain. The AP2 subfamily is characterized by two tandem AP2 domains, although a small number of proteins in the AP2 subfamily have only one AP2 domain. The RAV subfamily is much smaller than the other two subfamilies and is characterized by the presence of one AP2 domain and one B3 domain. In recent years, some AP2/ERF members have been assigned to another subfamily, soloist. Members of the soloist subfamily have significantly different aa sequences and gene structures than members of the ERF and AP2 subfamilies (Zhuang et al., 2011; Feng et al., 2020).

In addition to the AP2 domain, some AP2/ERFs also contain conserved activation or repression domains that affect downstream regulation of gene expression. The ERF-associated amphiphilic repression (EAR) motif (^L/F^DLN^L/F^(x)P) was the first repressor domain to be confirmed in the AP2/ERF family; it is present in the C-terminal of some AP2/ERF transcription factors (Ohta et al., 2001; Licausi et al., 2013). ^R/K^LFGV is another repressor domain found in the B3 domain of members of the RAV subfamily (Hiratsu et al., 2003; Ikeda and Ohme-Takagi, 2009), and EDLL is a strong acidic-type activation domain (Tiwari et al., 2012).

AP2/ERF transcription factors form one of the largest transcription factor families in plants and are key components in downstream ethylene signal transduction (Franco-Zorrilla et al., 2014); they regulate plant growth, development, stress responses, and other biological processes. In recent years, many studies have shown that AP2/ERF superfamily members are extensively involved in fruit development and ripening by affecting ethylene synthesis, chlorophyll degradation, coloring, fruit softening, and flavor formation (Zhai et al., 2022).

AP2/ERFs are involved in fruit ripening through regulation of the ethylene biosynthesis-related genes *1-aminocyclopropane-1-carboxylate oxidase* (*ACO*) in apple and pear and *1-aminocyclopropane-1-carboxylate synthase* (*ACS*) in banana and apple (Xiao et al., 2013; Han et al., 2016; Li et al., 2016; Hao et al., 2018). AP2/ERFs also participate in chlorophyll degradation, acting as transcriptional activators through binding to the promoters of chlorophyll degradation-related genes in apple (Yin et al., 2016; Han et al., 2018). AP2/ERFs in pear reportedly function together with myeloblastosis (MYB) and basic helix-loop-helix (bHLH) transcription factors to influence anthocyanin accumulation (Yao et al., 2017; Ni et al., 2019; An et al., 2020). AP2/ERFs regulate fruit softening by changing the expression of cell wall-related genes, such as *expansin*, *polygalacturonase*, *xyloglucan endotransglucosylase/hydrolases* (*XTHs*), *pectate lyase*, and *pectinesterase* in banana; *polygalacturonase* in peach; *polygalacturonase* and *pectinesterase* in papaya; and *XTH* in persimmon and kiwifruit (Yin et al., 2010; Fan et al., 2016; Fu et al., 2016; Han et al., 2016; Wang et al., 2017; Wang et al., 2019). Moreover, AP2/ERFs are involved in the synthesis and accumulation of many specialized metabolites; for example, they regulate the expression of genes related to aroma formation, such as *branched-chain amino acid transaminase* and *pyruvate decarboxylase* in banana (Feng et al., 2016) and *2-methylene-furan-3-one reductase* in strawberry (Zhang et al., 2018).

Fig (*Ficus carica*) originated in the Mediterranean area and was one of the earliest domesticated fruit trees. It is an important species, with both dry and fresh fruits eaten worldwide. Fig fruits have significant health value due to their antioxidant properties (Solomon et al., 2006). Fruit growth follows a sigmoidal curve, with stage I characterized by rapid increases in fruit size and weight, stage II having a long lag phase, and stage III characterized by superfast ripening over a very short duration, typically three to seven days. This is substantially shorter than the ripening phase of other common Mediterranean fruits, such as grapes, olives, and pomegranates. During stage III, fig fruit size and weight increase significantly and there is rapid sugar accumulation and fruit softening (Freiman et al., 2015; Kuang et al., 2022).

Fig was initially reported as a climacteric fruit (Marei and Crane, 1971), although in recent years the flesh and receptacle have been described as climacteric and non-climacteric, respectively (Freiman et al., 2015; Lama et al., 2019). Application of ethylene to fig fruits during stage II can accelerate fruit entry into stage III, promoting fig fruit ripening (Cui et al., 2021). Figs are dioecious, and the common female type can bear fruits by parthenocarpy or pollination (Flaishman et al., 2008). In contrast to parthenocarpic fruits, pollinated fruits are larger in diameter and weight, with improved firmness and a more commercially desirable appearance. During storage, senescence and spoilage are slower in pollinated fruits than in parthenocarpic fruits (Rosianski et al., 2016b).

Because fig fruits undergo superfast ripening that can be promoted by ethylene, it has been hypothesized that *AP2/ERF*s play important roles in fig fruit ripening. However, the *AP2/ERF* members present in fig and their expression patterns during fruit development have remained largely unknown. In this study, genome-wide identification of *AP2/ERF* genes was carried out in fig, and the gene structures, motif compositions, and chromosomal positions were determined. To investigate the relationship between fig fruit ripening and *AP2/ERF* expression, three transcriptomic datasets were used to analyze the expression patterns of *AP2/ERF* genes in fig fruits under several conditions: at different development stages; with and without ethephon treatment; and in parthenocarpic and pollinated fruits. This is the first genome-wide identification and expression pattern analysis of ethylene transcription factors in fig. This study revealed the most active *AP2/ERF* genes in fruit ripening, providing a critical reference for understanding the superfast ripening characteristics and quality formation of fig fruits. The results are valuable for future gene function mining studies and gene editing-assisted breeding.

## Materials and methods

### Physiological parameters of superfast fig fruit ripening

Five-year-old common figs (*F. carica* var. ‘Brown Turkey’) were used in this study. The trees had been planted from cuttings in the experimental station of China Agricultural University, Beijing, with 3 × 3 m spacing and a vertical trellis system. New shoots were managed with standard hedge training. Fruit ripened sequentially from the bottom to the top of each branch, meaning that fruits at similar heights were in the same developmental stages. At ~10 d before harvest, 32 fruits of different developmental stages were labeled, and the transverse diameter was measured with a vernier caliper every day. Three fruits were harvested every other day to measure fruit texture with a firmness meter (Mitutoyo GY-1 and 3, Japan) and soluble solid content with a refractometer (Atago PAL-1, Japan). There were three technical replicates of each measurement for each fruit.

### Identification of *AP2/ERF* gene family members in *F. carica*

*F. carica* genomic data were downloaded from NCBI (DDBJ/EMBL/GenBank access code: VYVB01000000) (Usai et al., 2020). *Arabidopsis thaliana* AP2/ERF protein sequences were downloaded from TAIR (https://www.arabidopsis.org). Using AtAP2/ERFs as the query sequences, a preliminary search was performed for fig *AP2/ERF* genes using BLASTP through Tbtools (E-value threshold ≤ 1e-5) (Chen et al., 2020). The Hidden Markov Model (HMM) file for the AP2 domain (PF00847) was downloaded from the Pfam database (http://pfam.xfam.org/), and sequences containing the AP2 domain were retrieved from the fig genome database using HMMER 3.0 (Finn et al., 2011). The results of the two screening methods were combined and redundant gene sequences removed. NCBI Batch-CD analysis confirmed that all resulting gene sequences contained the AP2 domain.

### Phylogenetic tree construction and AP2/ERF sequence analysis

All fig and Arabidopsis AP2/ERF protein sequences were aligned with ClustalW (Thompson et al., 2003), then a phylogenetic tree was constructed with MEGA11 using the maximum likelihood (ML) method with the following parameters: test of phylogeny, bootstrap method; number of bootstrap replicates, 1000; substitution type, amino acid; model/method, Jones-Taylor-Thornton (JTT) model; rates among sites, uniform rates; gaps/missing data treatment, use all sites; ML heuristic method, nearest-neighbor-interchange (NNI). Sequence length, molecular weight, and isoelectric point (pI) were computed with ProtParam (https://web.expasy.org/protparam/). The conserved motifs in AP2/ERF proteins were determined using MEME (http://meme.nbcr.net/meme/intro.html). Finally, gene structure was visualized with TBtools (Chen et al., 2020). The *FcAP2/ERF* promoters (the 2000-bp regions upstream of the start codon of each gene) were extracted from the fig genome and submitted to the PlantCare database (http://bioinformatics.psb.ugent.be/webtools/plantcare/html/) for identification of putative *cis*-regulatory elements.

### Chromosomal location and gene duplication

Chromosomal locations of fig *AP2/ERF* genes were determined using TBtools (Chen et al., 2020). Genomic data were obtained from http://plants.ensembl.org/index.html for *Vitis vinifera* and *Solanum lycopersicum* and from BIG Data Center (https://bigd.big.ac.cn/gsa/) for *Ficus hispida and Ficus microcarpa* (BioProject Accession number GSA: PRJCA002187) (Zhang et al., 2020). Interspecific and intraspecific syntenic analyses were performed with the Multiple Collinearity Scan toolkit (Wang et al., 2012). KaKs Calculator 2.0 was used to calculate the nonsynonymous substitution rate (Ka) to synonymous substitution rate (Ks) ratios (Wang et al., 2010). The divergence times in millions of years ago (Mya) were calculated as follows (Lynch and Conery, 2000): T = Ks/(2 × 6.1 × 10^-9^) ×10^-6^.

### *AP2/ERF* expression analysis in *F. carica* during fruit ripening

Expression levels of *AP2/ERF* genes were analyzed in the peel and flesh of ‘Purple Peel’ fig fruits during development by re-mining our previously sequenced and annotated transcriptome data (SRA accession: PRJNA723733) (Zhai et al., 2021). Briefly, six samples were taken during fruit development; samples 1 through 6 were taken at early stage I, mid stage I, early stage II, late stage II, mid stage III, and late stage III, respectively. Fruit peels (P) and flesh (F) were isolated and assayed separately at each timepoint (P1-P6 and F1-F6, respectively). There were three biological replicates for each sample. The RNA-Seq data generated from samples were matched to our laboratory’s previous transcriptome database using RSEM (RNA-Seq by Expectation Maximization) software package (Chai et al., 2017). Expression patterns were analyzed for *AP2/ERF* genes expressed at levels ≥ 20 fragments per kilobase of transcript per million mapped reads (FPKM) in at least one sample. If (sum_F)/(sum_P) was > 5 or < 0.2, a gene was defined as dominantly expressed in the flesh or peel, respectively. If (F4 + F5 + F6)/(F1 + F2 + F3) or (P4 + P5 + P6)/(P1 + P2 + P3) was > 2 or < 0.5, a gene was defined as positively or negatively correlated with fruit ripening, respectively.

The expression patterns of *AP2/ERF* genes in the fig fruit flesh and peel in response to ethephon treatment were analyzed by re-mining our previously sequenced and annotated transcriptome data (SRA accession: PRJNA606407) (Cui et al., 2021). Briefly, ‘Brown Turkey’ fig fruits in stage II were injected with 1 mL of 250 mg/L ethephon from the ostiole. Control and ethephon-treated fruits were collected at two, four, and six days after treatment (DAT), and fruit flesh and receptacle (R) transcriptomes were analyzed. There were three biological replicates of each sample. Annotation was conducted as described above for ‘Purple Peel’ samples. Gene expression patterns were analyzed for *AP2/ERF*s with values ≥ 20 transcripts per million (TPM) in at least one sample. Differentially expressed *FcAP2/ERF*s were classified as those with |log_2_(fold change) | ≥ 1 in an ethephon-treated sample compared to the control sample at the same timepoint in the same tissue.

*AP2*/*ERF* expression was also analyzed in pollinated and parthenocarpic ‘Brown Turkey’ fig fruits at two stages of development through re-mining transcriptome data submitted by the Flaishman group (SRA accession: PRJNA322124) (Rosianski et al., 2016a). Briefly, parthenocarpic (Par) and pollinated (Pol) fruits were collected at 60% and 100% ripeness (Par/Pol_60 and Par/Pol_100, respectively); RNA was extracted from the flesh and receptacles for sequencing and annotation. The raw sequencing reads were downloaded and mapped to the fig genome (DDBJ/EMBL/GenBank access code: VYVB01000000) using a series of plug-ins in TBtools, namely FastQC, Trimmomatic, and Kallisto (Chen et al., 2020). After obtaining a gene expression matrix, expression patterns were analyzed for *AP2/ERF*s that had FPKM values ≥ 20 in at least one sample. Differentially expressed *AP2/ERF*s were classified as those with |log_2_(fold change) | ≥ 1 in Par/Pol_100 compared to Par/Pol_60.

### Quantitative reverse transcription (qRT)-PCR validation of *AP2/ERF* gene expression during fruit development

Total RNA was extracted from ‘Purple Peel’ fruits at six developmental stages as described in our previous publications (Cao et al., 2016; Chai et al., 2017). cDNA was prepared with the PrimeScript RT Reagent Kit (Takara, Dalian, China) following the manufacturer’s instructions. *AP2/ERF* genes with relatively high expression levels were used for qRT-PCR validation, including RAV and ERF members, as well as genes with repression domains. Primer pairs for eight *AP2/ERF* genes were designed with Primer3 (https://bioinfo.ut.ee/primer3/). qRT-PCR was performed on an ABI QuantStudio 6 using TB Green^®^ Premix Ex Taq (RR420Q, Takara) with three technical replicates for each sample. The reaction conditions were as follows: 95°C for 30 s; 40 cycles of 95°C for 5 min and 60°C for 34 s. The 2^−△△CT^ method (Livak and Schmittgen, 2001) was used to determine relative gene expression using *elongation factor* (*c59932_g1*) as the internal control.

### Gene co-expression and protein interaction network analyses

Gene co-expression analysis and protein interaction network analysis were performed with Majorbio (https://cloud.majorbio.com). Spearmanʼs correlation coefficient was used to calculated gene co-expression. STRING (https://www.string-db.org) was used to generate the protein-protein interaction network based on the interaction network of homologs in *A. thaliana.* Connections were visualized in Cytoscape (Kohl et al., 2011).

## Results

### Superfast fig fruit ripening

**Figure 1:**
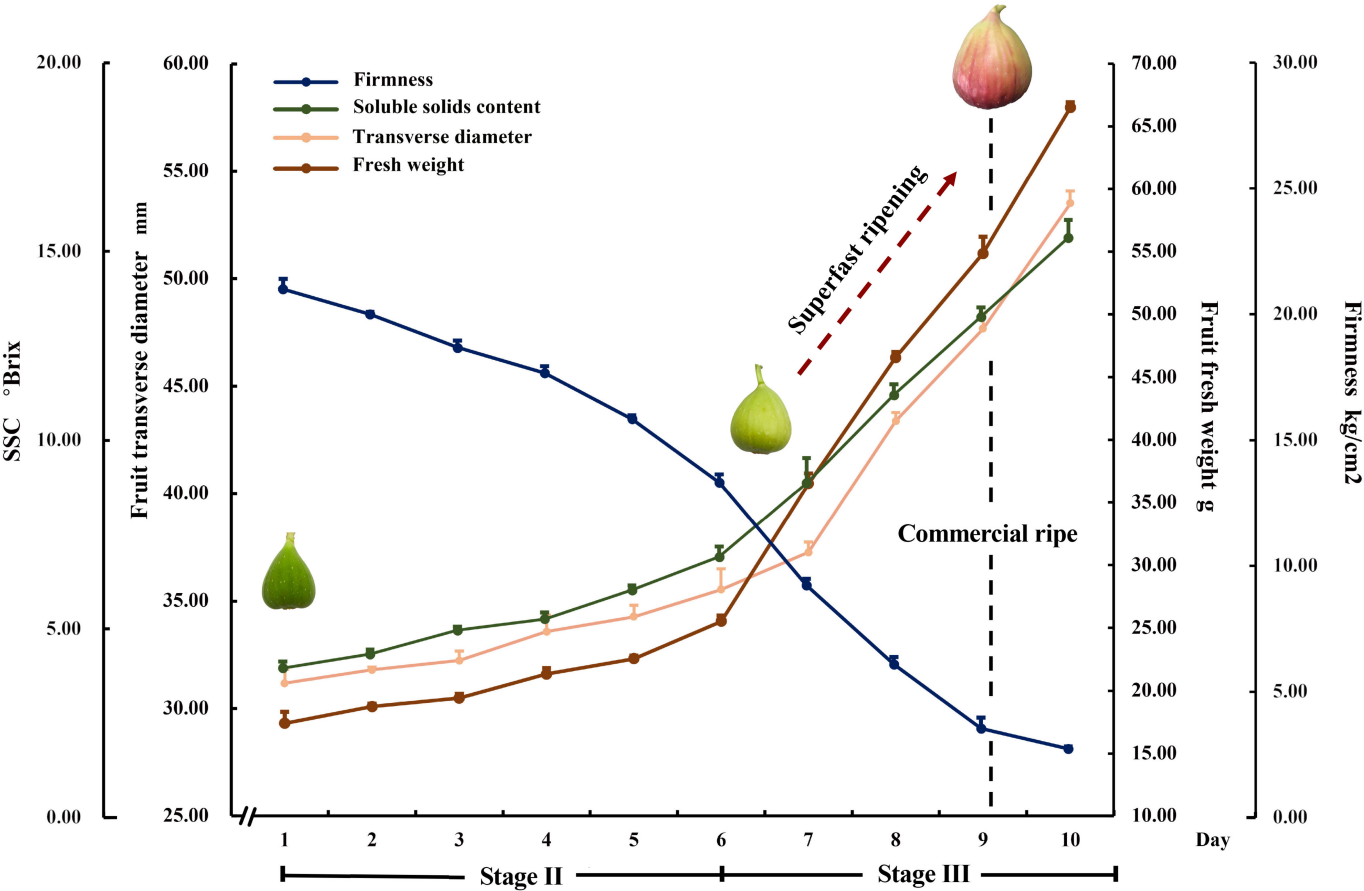
Superfast ripening in fig fruits.

Fig fruits showed very quick changes in major quality parameters during the last 10 d before they reached full ripeness. During the last four days, the transverse diameter and weight of fruits in stage III increased by an average of 4.49 mm and 10.24 g, respectively. The average increases in transverse diameter and weight per day for the last four days were 10.85% and 27.41%, respectively. This was a significant change compared to the average increases of 2.65% and 8.00% per day observed in late stage II. In addition, during the last four days, the soluble solid content increased by an average of 2.11°Brix (22.23% average increase per day) and the hardness decreased by an average of 2.65 kg/cm^2^ per day (32.33% average decrease per day). In contrast, there were only 11.80% average increases in soluble solid content and 8.60% average decreases in hardness per day in late stage II. Over two days (day 7 to day 9), the fruits reached commercial maturity; they were fully ripe one day later (**Figure 1**).

### *F. carica* AP2/ERF family member identification and gene structure

**Figure 2:**
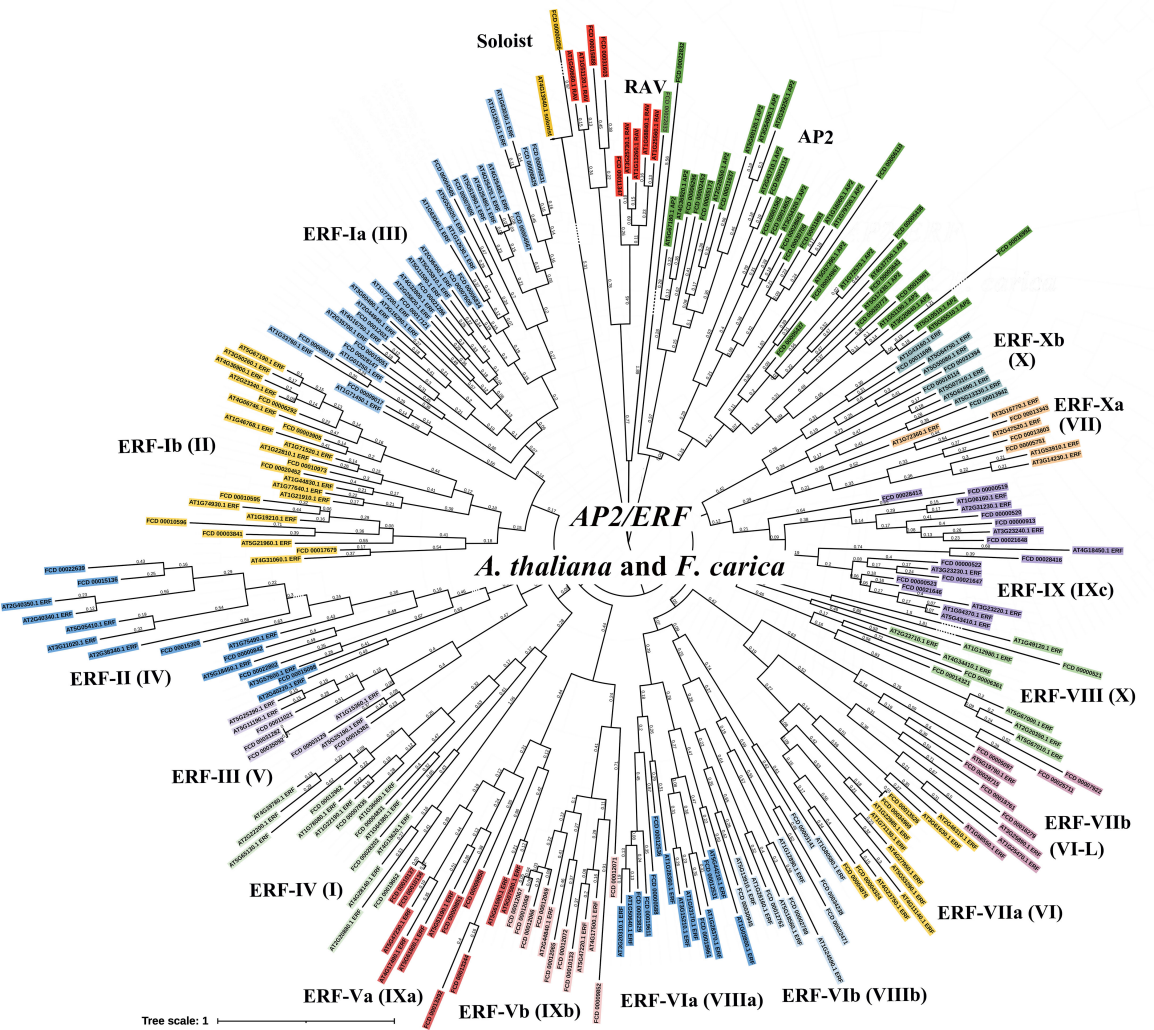
Phylogenetic tree showing the relationships between the 119 AP2/ERFs in *F. carica* and homologs in *A. thaliana*. There were 1000 bootstrap replicates.

**Figure 3:**
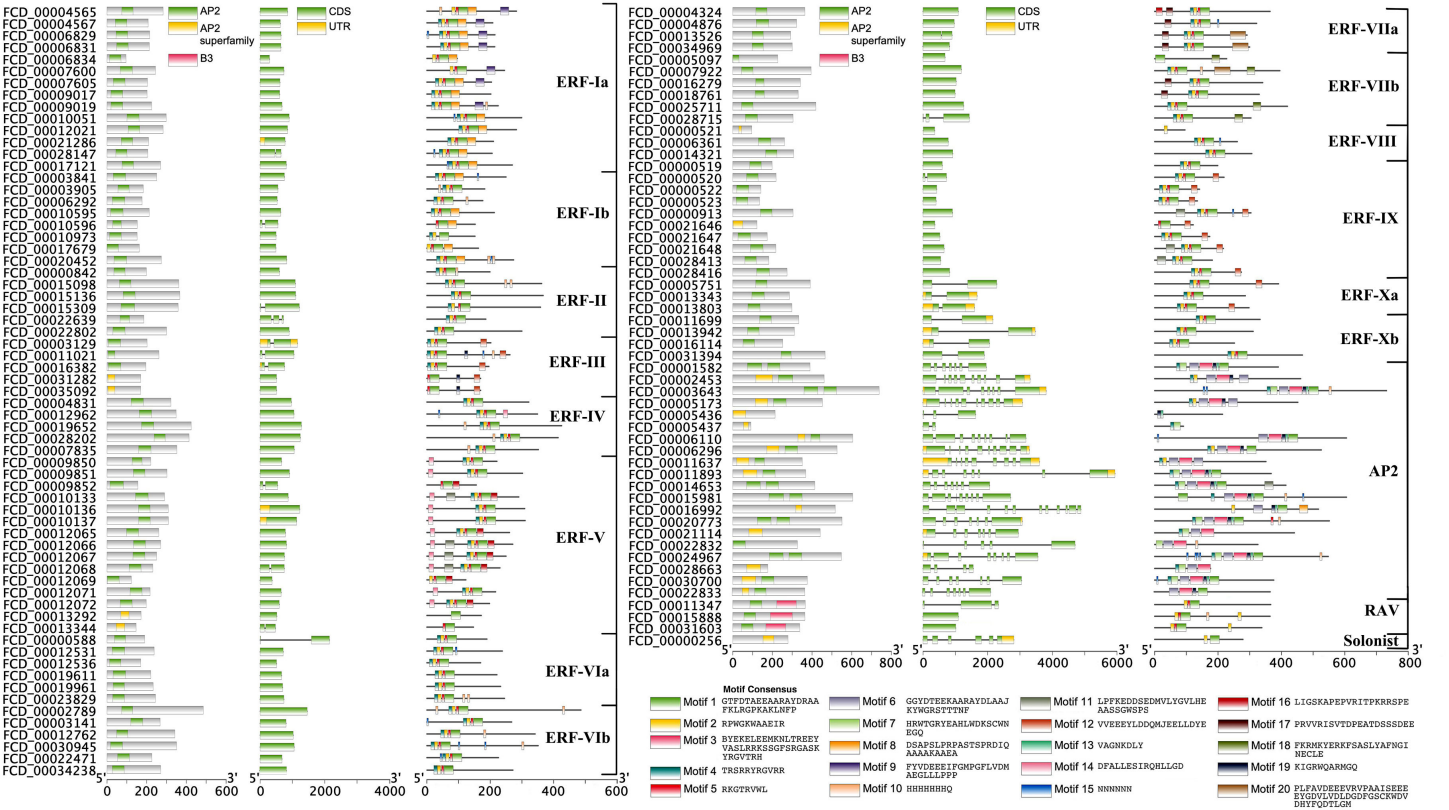
Analyses of domains (left), gene structure (middle), and motifs (right) in *F. carica* AP2/ERFs.

Putative fig *AP2/ERF* genes identified through Arabidopsis homologous gene alignment and Markov Model predictions were merged to remove redundant sequences. After NCBI Batch-CD analysis was performed to remove erroneous sequences, there were 119 unique candidate *AP2/ERF* genes in the fig genome. The aa sequence lengths of the encoded proteins ranged from 90-737, the protein molecular weights ranged from 10.0-80.4 kDa, and pI values ranged from 4.25-11.48 (**Figure 2** and **Supplementary Table 1**). Phylogenetic and sequence domain analysis revealed the presence of one *soloist* gene, 95 *ERF*s, 20 *AP2*s, and three *RAV* genes. Based on homology, the 95 *ERF*s were further divided into 15 subclasses (Ia, Ib, II, III, IV, Va, Vb, VIa, VIb, VIIa, VIIb, VIII, IX, Xa, and Xb) based on the classification of homologous genes in Arabidopsis (Nakano et al., 2006). Of the 20 AP2s, 14 contained two AP2 domains and six contained one AP2 domain (**Figures 2** and **3**).

Gene structure analysis showed that there were 76 *AP2/ERF* genes without introns, including 74 *ERF*s and 2 *RAV*s. Only 21 out of the 95 *ERF*s contained introns, of which two contained two introns and the rest contained only one intron. Interestingly, the AP2 subfamily generally had more introns—all 20 *AP2*s contained introns and 16 (80%) contained more than five introns. *FCD _00016992* contained the most introns (11) and exons (12) (**Figure 3**). Motif analysis demonstrated that most ERF subfamily members contained motifs 1, 2, 4, and 5; the 4-2-5-1 series was a feature of most ERF members. The majority of AP2 subfamily members contained motifs 3 and 6, which were unique to the AP2 subfamily. All three RAVs contained motifs 1, 2, and 5, and the soloist (FCD _00000256) contained only motifs 1 and 2 (**Figure 3**).

There were additional domains present in only a few of the AP2/ERFs. Four ERFs had EDLL activation domains in the C-terminal. Six ERFs and two AP2s contained the EAR repression domain. Two RAVs contained the R/KLFGV repression domain in the C-terminal. Significantly, both the middle and the C-terminal of one ERF (FCD_00012531) contained the EAR repression domain (**Supplementary Figure 1**).

### Chromosome distribution, collinearity, and synteny analysis

Of the 119 *AP2/ERF* genes identified, 116 were unevenly distributed across 13 chromosomes (**Supplementary Figure 2**). Three genes (one *RAV* and two *ERF*s) could not be located on any of the chromosomes (**Supplementary Table 2**). Fourteen *AP2/ERF* genes were located on the longest chromosome (chromosome 5), whereas there were only two on chromosome 6. Chromosomes 3 and 11 each contained two *RAV*s. Chromosomes 6, 9, and 12 had only *ERF* members (**Supplementary Figure 2**). Tandem duplication had occurred in *AP2/ERF* gene clusters located on chromosomes 4, 8, 9, 10, and 12. Phylogenetic analysis also showed clustering of tandem duplicates on those chromosomes (**Figure 3** and **Supplementary Figure 2**).

**Figure 4:**
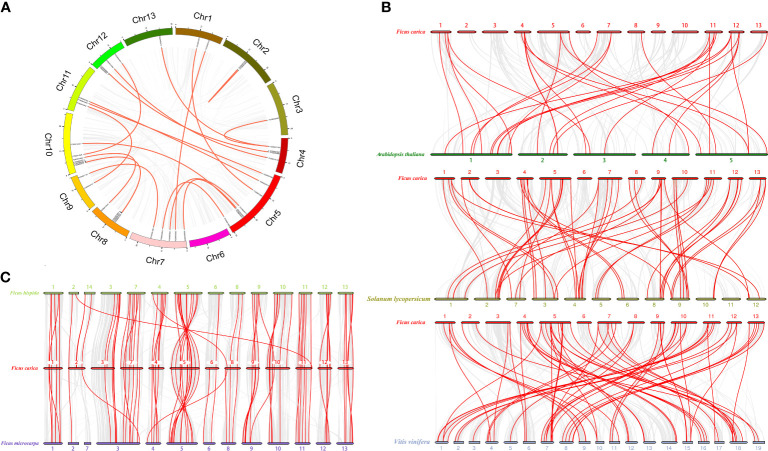
Collinearity and synteny analyses of *FcAP2/ERF* genes. **(A)** Collinearity of *FcAP2/ERF* genes. **(B)** Synteny analysis of *AP2/ERF* genes in *F. carica* and three other plant species. **(C)** Synteny analysis of *AP2/ERF* genes in three Ficus species (*F. carica*, *F. hispida*, and *F. macrocarpa*). Gray lines indicate all syntenic gene pairs within the genomes; red lines indicate syntenic *AP2/ERF* gene pairs.

A total of 215 collinear blocks were identified from analysis of collinearity among *AP2/ERF* genes in the fig genome. Twenty-one *FcAP2/ERF* genes, comprising four *AP2* and 17 *ERF* members, were unevenly distributed in 18 of these blocks (**Figure 4A**). Blocks 75 and 119 contained three and two *AP2/ERF* genes, respectively. The other blocks contained only one *AP2/ERF* gene each (**Supplementary Table 3**). Analysis of the Ka/Ks ratios revealed 32 pairs of paralogous *FcAP2/ERF* genes: 21 derived from segmental duplication and 11 from tandem duplication (**Supplementary Table 4**). The Ka/Ks ratios of the 32 gene pairs ranged from 0.05 to 0.57, suggesting that purifying selection was the primary force operating on these duplicate genes. The duplication events from which the 32 gene pairs were derived occurred between 1.81 and 379.01 Mya (**Supplementary Table 4**).

Syntenic analyses of *AP2/ERF* genes in fig, *A. thaliana*, *V. vinifera, S. lycopersicum*, *F. hispida*, and *F. macrocarpa* demonstrated that there was relatively high conservation of synteny between *F. carica* and *F. hispida* (83 orthologous gene pairs) (**Figures 4B, C**). There were 32, 58, 71, and 72 orthologous pairs between fig and *A. thaliana*, *V. vinifera, S. lycopersicum*, and *F. macrocarpa*, respectively (**Supplementary Table 5**). Sixteen *FcAP2/ERF*s were found in syntenic regions between all five species: one *RAV* (*FCD_00011347*), one *AP2* (*FCD_00006296*), and 14 *ERF*s (**Supplementary Figure 3A**), suggesting that these genes were highly evolutionarily conserved. Sixty-four *FcAP2/ERF*s (two *RAV*s, 16 *AP2*s, and 46 *ERF*s) were also found in syntenic relationships with both *F. hispida* and *F. macrocarpa* (**Supplementary Figure 3B**).

### Expression patterns during fruit ripening

**Figure 5:**
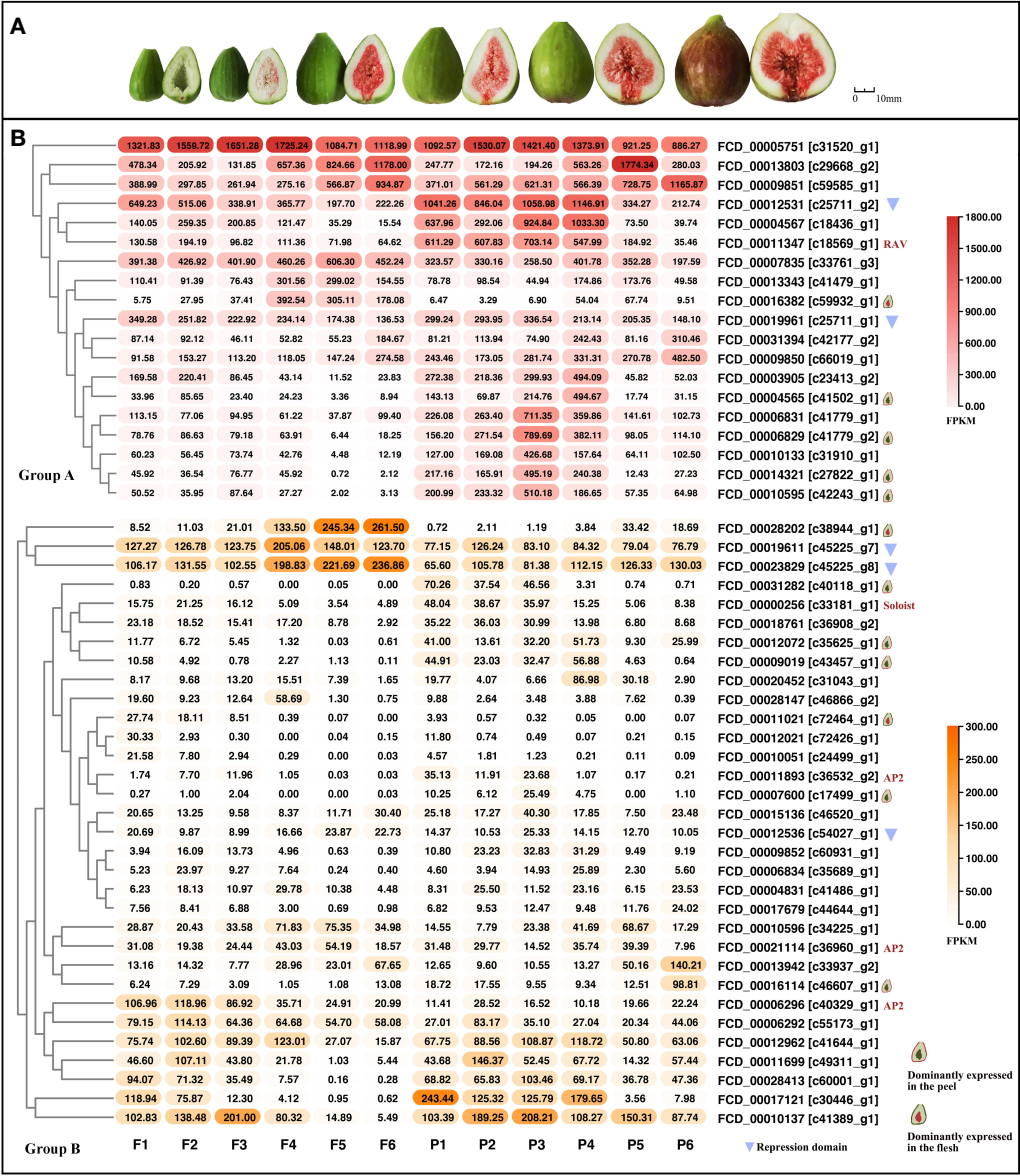
Expression patterns of *FcAP2/ERF* genes during fig fruit development. *FcAP2/ERF* genes were divided into three groups based on expression levels: group A (maximum FPKM between 300 and 1800), group B (maximum FPKM between 20 and 300) and group C (maximum FPKM ≤ 20). **(A)** Six stages of fig fruit development. **(B)** Expression patterns of genes in groups A and **(B)** Members of group C are shown in **Supplementary Figure 3**.

Gene expression levels were next analyzed for *AP2/ERF*s using previously generated ‘Purple Peel’ transcriptomes. There were 83 *FcAP2/ERF*s (namely 68 *ERF*s, 13 *AP2s*, one *RAV*, and one *soloist* gene) expressed in the flesh and peel samples at six developmental timepoints (**Figure 5** and **Supplementary Figure 4**). Genes were divided into three groups based on expression level: group A (containing genes with maximum FPKM values from 300-1800), group B (maximum FPKM from 20-300) and group C (maximum FPKM ≤ 20). There were 19, 32, and 32 *AP2/ERF* genes in groups A, B, and C, respectively. Group A consisted of one *RAV* and 18 *ERF* genes; group B contained one *soloist*, three *AP2*, and 28 *ERF* genes; and group C contained 10 *AP2*s and 22 *ERF*s. Most of the *AP2* genes had relatively low FPKM values and were therefore in group C (**Figure 5B** and **Supplementary Figure 4**).

*AP2/ERF*s with FPKM values ≥ 20 in at least one of the 12 samples (two tissues at six timepoints) were further analyzed. Genes were categorized as being dominantly expressed in either the flesh or peel if they had a greater than five-fold difference in the sum of FPKM values for all samples from either the flesh or the peel tissue, respectively. There were nine *FcAP2/ERF*s dominantly expressed in the peel and three in the flesh (**Figure 5B** and **Supplementary Table 6**). *FCD_00007600 [c17499_g1]* was the most dominantly expressed *ERF* in the flesh; when the FPKM values were summed across all samples, it was expressed 14.28 times more highly in the flesh than in the peel. Furthermore, this gene was conserved among the three Ficus species studied here. *FCD_00031282* [*c40118_g1*] was the most dominantly expressed *ERF* in the peel, with the summed expression being 96.44 times higher in the peel than in the flesh (in which the FPKM was < 1 at all six stages) (**Supplementary Table 6**). This *ERF* was shown to have homologs in *S. lycopersicum* and *F. hispida* (**Supplementary Table 5**).

**Figure 6:**
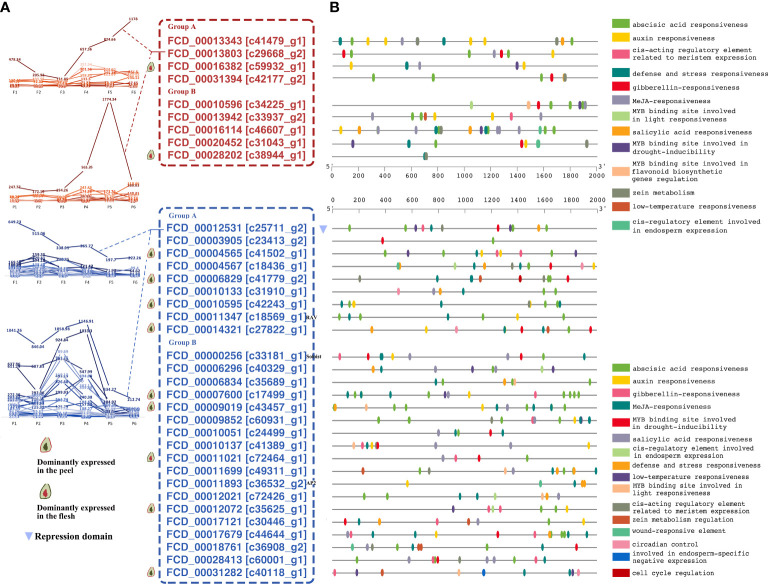
*FcAP2/ERF* genes significantly correlated with fruit ripening and analysis of their promoter regions. The thresholds used were FPKM ≥ 20 in at least one sample and the sum of expression values on days four through six divided by the sum of expression values on days one through three > 2 or < 0.5. **(A)** *FcAP2/ERF* genes positively or negatively correlated with fruit ripening. **(B)** Promoter region analysis of *FcAP2/ERF* genes significantly correlated with fruit ripening.

Nine *ERF*s were found to be positively correlated with fruit ripening; in contrast, 27 *AP2/ERF*s (one *RAV*, one *AP2*, one *soloist*, and 24 *ERF*s) were negatively correlated with fruit ripening (**Figure 6A**). Of the genes significantly correlated with fruit ripening, eight *ERF*s were dominantly expressed in the peel, all of which were negatively correlated with fruit ripening. Three *ERF*s were dominantly expressed in the flesh and had similar expression patterns; one was positively correlated and two were negatively correlated with fruit ripening. Although there were large differences in the expression levels of these genes between the flesh and peel, the expression patterns were comparable (**Figure 6A**). The FPKM values of *FCD_00013803* [*c29668_g2*] and *FCD_00012531* [*c25711_g2*] were expressed at extremely high levels. The former was positively correlated with ripening and was conserved among the three Ficus species, whereas the latter was negatively correlated with ripening and was conserved among all five species used in the syntenic analysis (**Figure 6A** **Supplementary Figure 3**). The *Arabidopsis* homolog of *FCD_00013803*, *AtERF73*, functions as a transcriptional activator in the hypoxia response and in root development (Seok et al., 2014). *FCD_00012531* contained two EAR repression motifs (**Supplementary Figure 1**). Tobacco (*Nicotiana tabacum*) contains a homolog of *FCD 00012531*, *NtERF4*, which also has an EAR repression motif and acts as a transcriptional repressor (Ohta et al., 2000).

Analysis of the promoter regions showed that there were no significant differences in the type or number of promoter elements between genes positively and negatively correlated with fruit ripening. The main elements in the promoters of *FcAP2/ERF*s were hormone-related and abscisic acid (ABA) response-related elements (**Figure 6B**), demonstrating the importance of ABA and ethylene in fig fruit ripening and suggesting crosstalk between the two pathways. Four hormone-related elements, namely responsiveness to ABA, auxin, gibberellin, and methyl jasmonate (MeJA), were present in the promoter region of *FCD_00013803*[*c29668_g2*], which was significantly positively correlated with ripening. The promoter of *FCD_00012531*[*c25711_g2*], which was significantly negatively correlated with ripening, contained elements related to both hormones (ABA and gibberellin) and stress resistance (e.g., low temperature and defense responses) (**Figure 6B**).

Eight *FcAP2/ERF* genes were selected for qRT-PCR verification of the ‘Purple Peel’ fruit ripening RNA-Seq results (**Supplementary Table 7**). The expression patterns observed *via* qRT-PCR were similar to those seen in the RNA-Seq results (R^2^ = 0.72) (**Supplementary Figure 5**).

### Ethephon treatment altered *AP2/ERF* gene expression patterns in fruits

**Figure 7:**
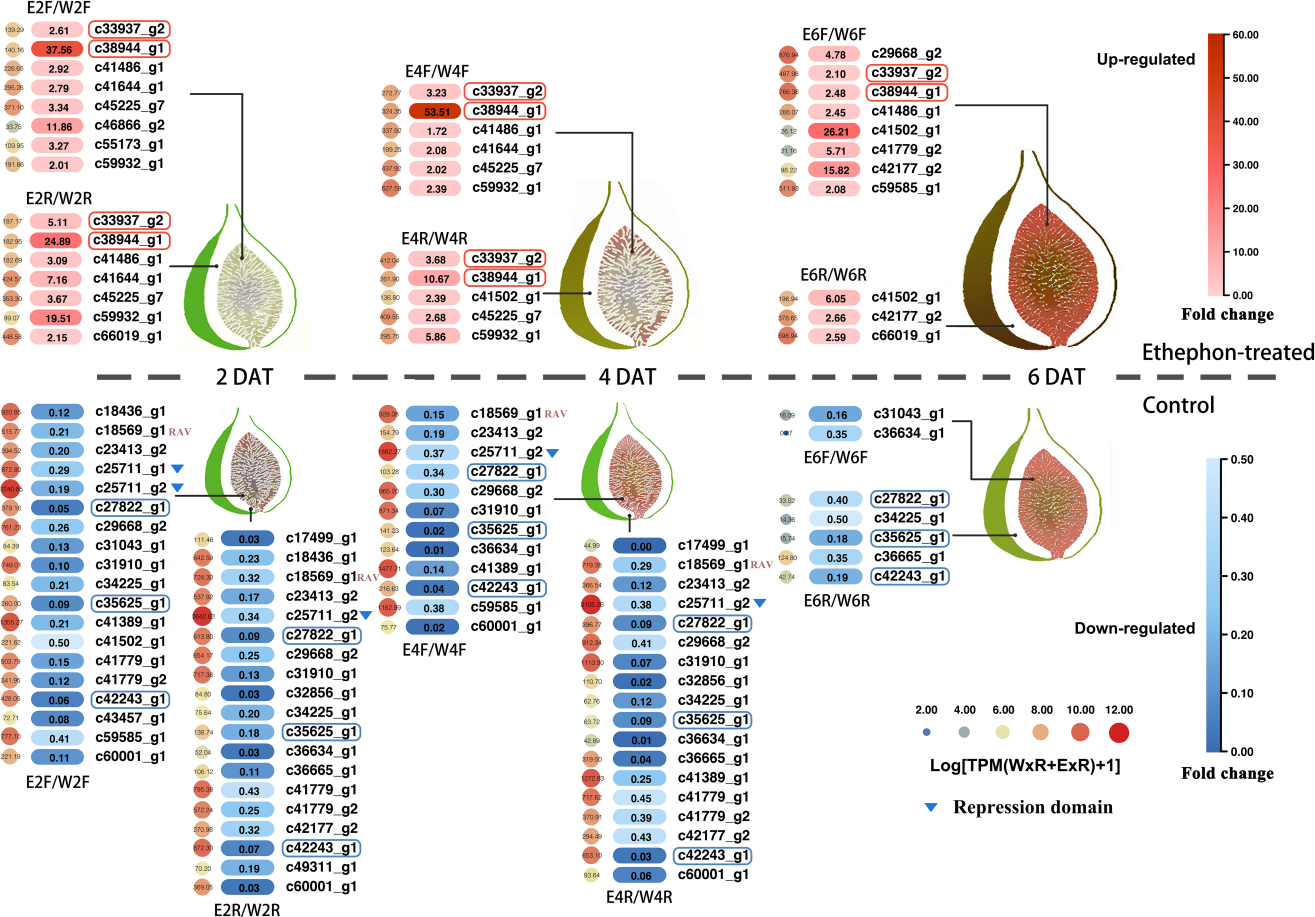
Changes in *FcAP2/ERF* gene expression in fig fruit flesh and receptacle tissues after ethephon treatment. Color intensity indicates the ratio of expression in ethephon-treated tissues compared to control tissues. Genes circled in red or blue were differentially expressed in all five comparisons of treated to control tissues.

Treatment with ethephon caused fig fruits in stage II to ripen six days earlier than control fruits. Fruits treated with ethephon were larger and softer and pigmented faster. After removing lowly-expressed genes, 34 expressed *FcAP2/ERF* genes were identified, one *RAV* (*FCD_00011347* [*c18569_g1*]) and 33 *ERF*s, with ≥ two-fold changes in expression in the flesh or receptacle after ethephon treatment (**Figure 7** and **Supplementary Table 8**).

Differentially expressed genes were then analyzed in the flesh (F) and receptacle (R) of ethephon-treated fruits (E) compared to the controls (W) on each sampling day (two, four, and six) for a total of six comparisons: E2F vs. W2F; E2R vs. W2R; E4F vs. W4F; E4R vs. W4R; E6F vs. W6F; and E6R vs. W6R. There were eight, seven, six, five, eight, and three *AP2/ERF* genes upregulated and 19, 19, 12, 18, two, and five *AP2/ERF* genes downregulated in E2F, E2R, E4F, E4R, E6F, and E6R, respectively. There were more *FcAP2/ERF*s downregulated than upregulated at days two and four after ethephon treatment (**Figure 7**). Two *ERF* genes (*FCD_00013942* [*c33937_g2*] and *FCD_00028202* [*c38944_g1*]) were upregulated in E2F, E2R, E4F, E4R, and E6F. The gene *c38944_g1* was most highly upregulated in E2F and E4F, at 37.56 and 53.51 times, respectively (**Figure 7**). Three *ERF* genes (*FCD_00014321 [c27822_g1]*, *FCD_00012072 [c35625_g1]*, and *FCD_00010595* [*c42243_g1*]) were downregulated in E2F, E2R, E4F, E4R, and E6R. Two *ERF* genes (*FCD_00019961* [*c25711_g1*] and *FCD_00012531* [*c25711_g2*]), which contained EAR repression domains, were downregulated in E2F, and the latter was also downregulated in E4F, E2R, and E4R. The *RAV* gene (*FCD_00011347* [*c18569_g1*]) was downregulated in response to ethephon in E2F, E2R, E4F, and E4R, suggesting a repressive role of these genes in fruit ripening (**Figure 7**).

### *AP2/ERF* expression patterns during pollinated and parthenocarpic fruit ripening

**Figure 8:**
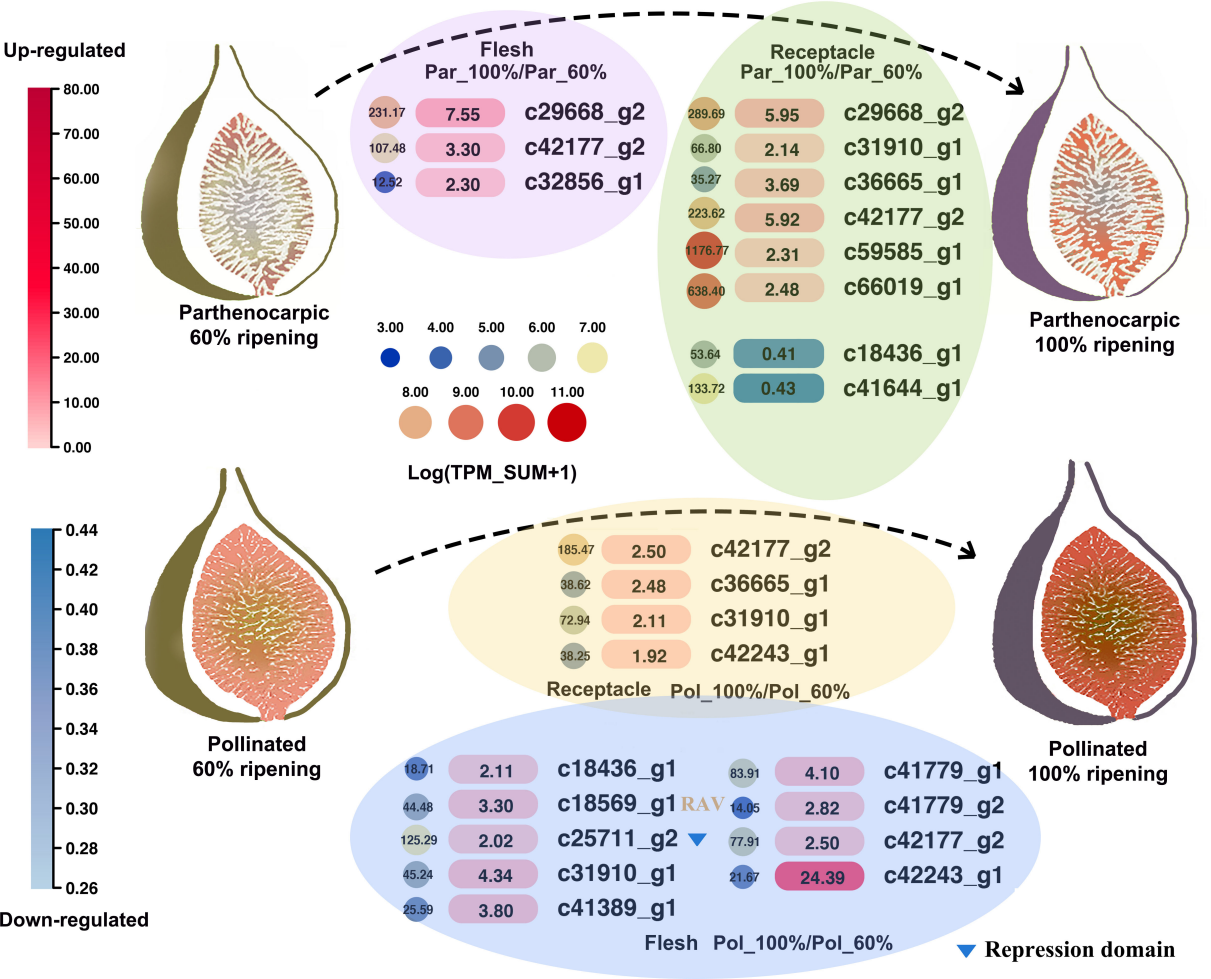
Changes in *FcAP2/ERF* gene expression between fig fruit flesh and receptacles at 60% and 100% ripeness in pollinated and parthenocarpic fruits. Color intensity indicates the ratio of expression in 100% compared to 60% ripe tissues.

Gene expression was next compared between pollinated and parthenocarpic fruits in a total of four conditions: flesh and receptacle samples each from fruits at 60% and 100% ripeness (**Figure 8**). Fifteen differentially expressed *FcAP2/ERF* genes were identified using threshold values of TPM ≥ 20 in at least one of the eight samples and |log_2_(fold change) | ≥ 1 in fruits at 100% ripeness compared to 60% ripeness. One *RAV* gene (*FCD_00011347* [*c18569_g1*]) and 11 *ERF*s differentially expressed between 100% and 60% ripeness were also differentially expressed in response to ethephon treatment (**Figure 8**). Interestingly, most *FcAP2/ERF*s were upregulated at 100% compared to 60% ripeness. There were only two downregulated *ERF*s, *FCD_00004567* [*c18436_g1*] and *FCD_00012962* [*c41644_g1*], in 100% vs. 60% ripe parthenocarpic fruit receptacles; these were also downregulated during ‘Purple Peel’ fig fruit development and were conserved among all five plant species included in the syntenic analysis (**Figure 8** and **Supplementary Figure 3**). The *RAV* gene (*FCD_00011347* [*c18569_g1*]) was upregulated in the flesh of 100% ripe compared to 60% ripe pollinated fruits. *FCD_00031394* [*c42177_g2*] was upregulated in 100% ripe compared to 60% ripe fruits, in both parthenocarpic and pollinated fruit flesh and receptacles. In the ethephon treatment data, *FCD_00031394* [*c42177_g2*] was also found to be upregulated during ripening and at the late ripening stage (E6F vs. W6F and E6R vs. W6R) (**Figures 6** and **7**). Thus, *c42177_g2* may be a positive regulator of fruit ripening.

### Potential key *FcAP2/ERF*s in superfast fruit ripening

**Figure 9:**
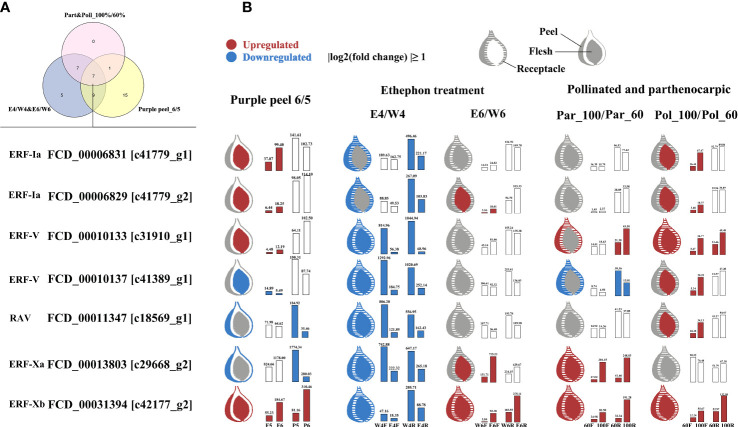
Summary of expression patterns for seven key *FcAP2/ERF* genes identified as differentially expressed in three transcriptomic datasets. **(A)** The intersection of *FcAP2/ERF*s differentially expressed in all three transcriptomic datasets. **(B)** Fruit ripening comparisons. The bar graph shows gene expression values in FPKM or TPM.

To identify *FcAP2/ERF*s associated with superfast fig fruit ripening, multiple datasets were integrated and genes that were consistently differentially regulated at the superfast ripening stage were screened. For each condition, comparisons were made by calculating the ratio of gene expression in the riper compared to the less ripe fig fruit. Specifically, the samples compared were F6/F5 and R6/R5 in ‘Purple Peel’ and E4/W4, E6/W6, Pol_100/Pol_60, and Par_100/Par_60 in the flesh and receptacles of ‘Brown Turkey’. Six *ERF*s (from the ERF-I, -V, and -X subgroups) and one *RAV* were identified as differentially regulated in all datasets (**Figure 9A**).

These seven consistently differentially regulated *FcAP2/ERF*s were all downregulated four days after ethephon treatment (**Figure 9B**). *FCD_00006829 [c41779_g2]* and *FCD_00031394* [*c42177_g2*] were upregulated in the flesh, peel, and receptacle during fig fruit ripening at levels between 1.02 times (receptacles of Pol_100 vs. Pol_60) and 15.82 times (E6F vs. W6F). These two *ERF*s may therefore be activators of fig fruit ripening. *FCD_00010137 [c41389_g1]* and *FCD_00011347 [c18569_g1]* were downregulated in the flesh, peel, and receptacle during ‘Purple Peel’ fruit ripening and in ethephon-treated and parthenocarpic ‘Brown Turkey’ fruits; these genes may therefore be repressors of fruit ripening. The level of downregulation was between 0.14 times (E4F vs. W4F) and 0.96 times (flesh of Par_100 vs. Par_60). Pollination also altered expression of the two *ERF*s; they were upregulated in the flesh and receptacle from 60% to 100% ripeness in pollinated figs (**Figure 9B**).

Arabidopsis homologs of the seven consistently differentially expressed *FcAP2/ERF*s were identified and interacting proteins were screened (**Supplementary Table 9**). The most striking result was observed for the homolog of *FcRAV* (*FCD_00011347* [*c18569_g1*]), which was considered to be a repressor because it was downregulated during fruit ripening in ‘Purple Peel’ and after ethephon treatment in ‘Brown Turkey’ (**Figures 6** and **7**). The homolog, *AT1G13260*, interacted with Topless-Related proteins (TPRs), Highly ABA-Induced (HAI1), and Sucrose Nonfermenting 1-Related Protein Kinases (SNRKs). TPRs always act as transcriptional co-repressors (Xu et al., 2021), suggesting that the *RAV* gene in fig may inhibit fruit ripening by recruiting transcriptional co-repressors. HAI1 and SNRKs are involved in the ABA signaling network (Chong et al., 2019; Shang et al., 2022), indicating that the *RAV* gene may be regulated by both ethylene and ABA.

## Discussion

In this study, genome-wide identification and gene structure analyses were carried out for AP2/ERF gene family members in *F. carica* for the first time. Due to the extensive involvement of *AP2/ERF*s in fruit ripening, three transcriptomic datasets related to fig fruit ripening were used to identify *FcAP2/ERF* genes expressed in fig fruits and to measure their expression patterns. Our findings provide new insights into the expression patterns and possible functions of *FcAP2/ERF*s and establish promising candidate fruit ripening-related *FcAP2/ERF*s for further study.

### Evolution of the AP2/ERF family

Gene duplication plays an important role in plant evolution. Homologous genes are generated by mechanisms such as tandem and segmental duplication, which form the basis for the emergence of new genes and novel functions (Birchler and Yang, 2022). The 119 *FcAP2/ERF* genes identified in the fig genome were found to have been derived from tandem and segmental duplication events. Furthermore, syntenic analysis with *A. thaliana*, *V. vinifera, S. lycopersicum*, *F. hispida*, and *F. macrocarpa* showed that 16 of the *FcAP2/ERF* genes had homologs in all five species (**Supplementary Figure 3**). These 16 *FcAP2/ERF* genes therefore appeared to be evolutionarily conserved and may have existed in a common ancestor. Although the origin of the *AP2/ERF* family in plants is uncertain, it has been speculated that it resulted from the transfer of an HNH-AP2 endonuclease gene from bacteria or viruses into plants (Magnani et al., 2004).

Significantly, *FcAP2* subfamily members contained far more introns than other *AP2/ERF* subfamilies in fig. Eukaryotic genes can be classified as intron-less (no introns), intron-poor (three or fewer introns) or intron-rich (more than three introns) (Liu et al., 2021). *FcAP2* subfamily members were found to be intron-rich (**Figure 3**). Studies have shown that intron loss is accelerated after gene fragment duplication (Lin et al., 2006). Intron-less and intron-poor genes were also shown to have evolved more recently and to be more functionally constrained than intron-rich genes (Liu et al., 2021). Therefore, it has been hypothesized that *FcERF*s and *FcRAV*s, which have fewer introns, evolved later than the *AP2* subfamily, and may in fact have been derived from the *AP2* subfamily to perform additional biological functions. This hypothesis was supported by the low expression levels of most *FcAP2* genes observed in this study (**Figure 5** and **Supplementary Figure 4**).

### Repressors in the AP2/ERF family

Analysis of two transcriptomic datasets related to fruit ripening showed that there were more downregulated than upregulated *FcAP2/ERF*s during fruit ripening (**Figures 6** and **7**), suggesting that those genes may serve as repressors. The EAR motif is the most prevalent repression motif that has been identified in plants (Kagale and Rozwadowski, 2011). Gene structure analysis showed that *FCD_00019611* and *FCD_00012531*, which were downregulated during fruit ripening (**Figure 6**), contained EAR motifs (**Supplementary Figure 1**). They were homologs of the Arabidopsis repressor genes *AtERF3* and *AtERF4* (**Supplementary Figure 1**), and therefore likely play transcriptional inhibitory roles in fig fruit development.

Interaction network analyses of AtERF3 and AtERF4 provided information about the possible functions of their fig homologs. AtERF3 and AtERF4 were experimentally proven to interact with SAP18. In addition, AtERF3, HD1, and SAP18 can interact with each other (**Supplementary Figure 6**). In yeast and mammalian systems, transcriptional downregulation involves core histone deacetylation, which results in compact nucleosome structure and thus suppresses gene expression. This process is mediated by a complex containing HDA1, SAP18, SAP30, and other proteins (Knoepfler and Eisenman, 1999). AtSAP18 has been shown to act as a linker, connecting the HDA complex to transcriptional repressors that are bound to chromatin in a sequence-specific manner, leading to transcriptional repression (Song and Galbraith, 2006). We therefore hypothesize that FcERFs with the EAR motif mediates transcriptional repression, possibly *via* histone deacetylation, in fig fruits.

### Plant hormones and AP2/ERFs

Plant hormones are well-known regulators of fruit ripening. In this study, many hormone-related elements were identified in the promoters of *FcAP2/ERF* genes (**Figure 6**). Our previous studies showed that plant hormones interfered with the expression of *FcAP2/ERF*s in fig fruits. After gibberellin treatment, members of the FcAP2/ERF family showed differing expression patterns (Chai et al., 2018). After cytokinin treatment, most *AP2/ERF*s were downregulated in fig flesh but upregulated in the receptacles (Chai et al., 2019). After ethephon treatment, most *AP2/ERF*s were downregulated in both flesh and receptacles (Cui et al., 2021). Moreover, ABA, auxin, MeJA, and brassinolide (BR) also mediate changes in plant growth and development through *AP2/ERF*s (Hu et al., 2004; Zhu et al., 2010; Gao et al., 2019; Lin et al., 2020). In turn, *AP2/ERF*s affect plant hormone synthesis. The most well characterized of these processes are *AP2/ERF* mediation of ethylene and ABA synthesis (Zhang et al., 2013; Sun et al., 2021), but jasmonate and auxin can also be regulated by *AP2/ERF*s (Blencowe et al., 2006; Tan et al., 2018; Xie et al., 2019). The involvement of AP2/ERFs in hormone signaling and synthesis adds to the complexity of the known plant hormone regulatory network. This elaborate regulatory mechanism improves the adaptability of plants to the environment and necessitates further exploration of the functions of important *FcAP2/ERF*s.

### AP2/ERFs are associated with fig fruit ripening

Figs undergo a very rapid ripening process, during which a synchronous peak in respiration rate and ethylene release has been reported (Marei and Crane, 1971). Ethylene has long been used in horticultural crop production to promote fruit ripening and to improve quality attributes such as pigmentation. In fig, ethylene treatment at stage II stimulates fruit growth and ripening; ethylene-treated figs ripen between four and 11 days earlier than untreated controls (Marei and Crane, 1971; Cui et al., 2021). Ethylene treatment promotes the synthesis of ribosomes, ribonucleic acids, and proteins in fig fruits (Marei and Crane, 1971) and induces upregulation of genes that are involved in biosynthesis of and responses to ethylene (Lama et al., 2018). By joint screening of three transcriptomic datasets derived from developing and ripening fig fruits, nine candidate *FcAP2/ERF* genes were identified based on expression levels and patterns consistent with fruit ripening.

The poor storability and short shelf lives of fig fruits are the main limitations in the development of the fresh fig industry. Rapid decreases in fruit hardness and loss of texture in the postharvest stage are tightly connected with the superfast ripening process. *ERF* subfamily genes have been shown to regulate fruit softening by changing the expression of cell wall-related genes, such as *ERF.B3* of *Solanum lycopersicum*, *ERF9* of *Actinidia chinensis*, *ERF11* of *Musa nana, ERF9* of *Chaenomeles sinensis, ERF8/16/19* of *Diospyros kaki*, and *ERF2* of *Amygdalus persica* (Gao et al., 2020). In previous studies, we identified genes associated with fig fruit softening, including *polygalacturonase*, *pectinesterase inhibitor, pectate lyase*, and *expansin* (Cui et al., 2019; Cui et al., 2021). AP2/ERF-binding motifs were found to be abundant in the fig *pectate lyase* promoter (**Supplementary Figure 7**), further in-depth studies are being conducted at present. With the rapid development and adoption of gene editing technologies in crop sciences, precise control of fruit softening without alteration of other important fruit quality attributes could be achieved by manipulating the expression of genes that specifically affect fruit softening. Understanding the functions of key *AP2/ERF*s in superfast fig fruit ripening will allow for selection of appropriate genes to achieve this goal.

## Conclusion

A total of 119 *AP2/ERF* genes were identified in the *F. carica* genome, namely 95 *ERFs*, 20 *AP2s*, three *RAVs* and one *soloist*. The evolutionary and expression pattern analyses conducted here provide valuable information about the evolution, characteristics, and fruit ripening-related functions of *FcAP2/ERF* genes. Multi-omics data allowed for screening of potential key *FcAP2/ERF* genes involved in fruit ripening. The results of this study provide valuable findings for further investigation and contribute to understanding of the roles of *AP2/ERF* genes in fig fruit development.

## Data availability statement

Publicly available datasets were analyzed in this study. This data can be found here: The RNA-Seq data used in this study derived from NCBI (SRA accession: PRJNA723733 [six stages], PRJNA606407 [ethephon treatment], and PRJNA322124 [pollinated and parthenocarpic]).

## Author contributions

YC, YZ, JH, and MS prepared the data. YC and YZ completed the analysis and conducted the experiments. YC, YZ, JH, MS, MF, and HM prepared the manuscript. All authors have read and approved the manuscript for publication.

## Funding

This work was supported by the key research project for fig development of Weiyuan County and National Natural Science Foundation of China project NSFC [31372007].

## Conflict of interest

The authors declare that the research was conducted in the absence of any commercial or financial relationships that could be construed as a potential conflict of interest.

## Publisher’s note

All claims expressed in this article are solely those of the authors and do not necessarily represent those of their affiliated organizations, or those of the publisher, the editors and the reviewers. Any product that may be evaluated in this article, or claim that may be made by its manufacturer, is not guaranteed or endorsed by the publisher.

## Supplementary material

Supplementary Table 1 Amino acid (aa) length, molecular weight (MW), and isoelectric point (pI) values for the proteins encoded by the 119 FcAP2/ERF genes.

Supplementary Table 2 The *FcAP2/ERF* genes that failed to align to any of the chromosomes.

Supplementary Table 3 Detailed information about intergenomic collinearity in the fig genome.

Supplementary Table 4 Divergence between paralogous *FcAP2/ERF* gene pairs in *F. carica*. S, segmental duplication; T, tandem duplication. Members of the AP2 family are shown in bold.

Supplementary Table 5 Synteny analysis of *AP2/ERF* gene pairs in *F. carica* and five other plant species.

Supplementary Table 6
*FcAP2/ERF* genes with a greater than five-fold change in the sum of expression values between the flesh and peel.

Supplementary Table 7 Primers used for quantitative reverse transcription (qRT)-PCR.

Supplementary Table 8 Detailed information about differentially expressed *FcAP2/ERF* genes in fig flesh and receptacle tissues in response to ethephon treatment.

Supplementary Table 9 Interactions between nine key FcAP2/ERF homologs in *A. thaliana*.

Supplementary Figure 1 EDLL activation domains, EAR repression domains, and ^R/K^LFGV domains in FcAP2/ERFs.

Supplementary Figure 2 Chromosomal distribution of *FcAP2/ERF*s. Red lines indicate *AP2/ERF* gene pairs derived from tandem duplication. The number after each chromosome name indicates the number of *FcAP2/ERF* genes mapped to that chromosome.

Supplementary Figure 3 Veen analysis of syntenic *AP2/ERF*s between *F. carica* and five other plant species. **(A)** *FcAP2/ERF* genes that showed syntenic relationships with all five plant species. **(B)** *FcAP2/ERF* genes that showed syntenic relationships with the other two Ficus species, *F. hispida* and *F. macrocarpa*.

Supplementary Figure 4 Expression patterns of lowly-expressed (group C) genes at six fruit developmental stages.

Supplementary Figure 5 Quantitative reverse transcription (qRT)-PCR validation of RNA-Seq results at six fruit developmental stages.

Supplementary Figure 6 Interaction networks of *FCD_00019611* and *FCD_00012531* based on those of *A. thaliana* homologs.

Supplementary Figure 7 AP2/ERF binding element in the promoter of *pectate lyase.*
