# Supplementary figures and images for: AP2/ERF genes associated with superfast fig (Ficus carica L.) fruit ripening
Source: Front Plant Sci. 2022 Oct 31;13:1040796. doi: 10.3389/fpls.2022.1040796 (PMC9659990; doi:10.3389/fpls.2022.1040796)

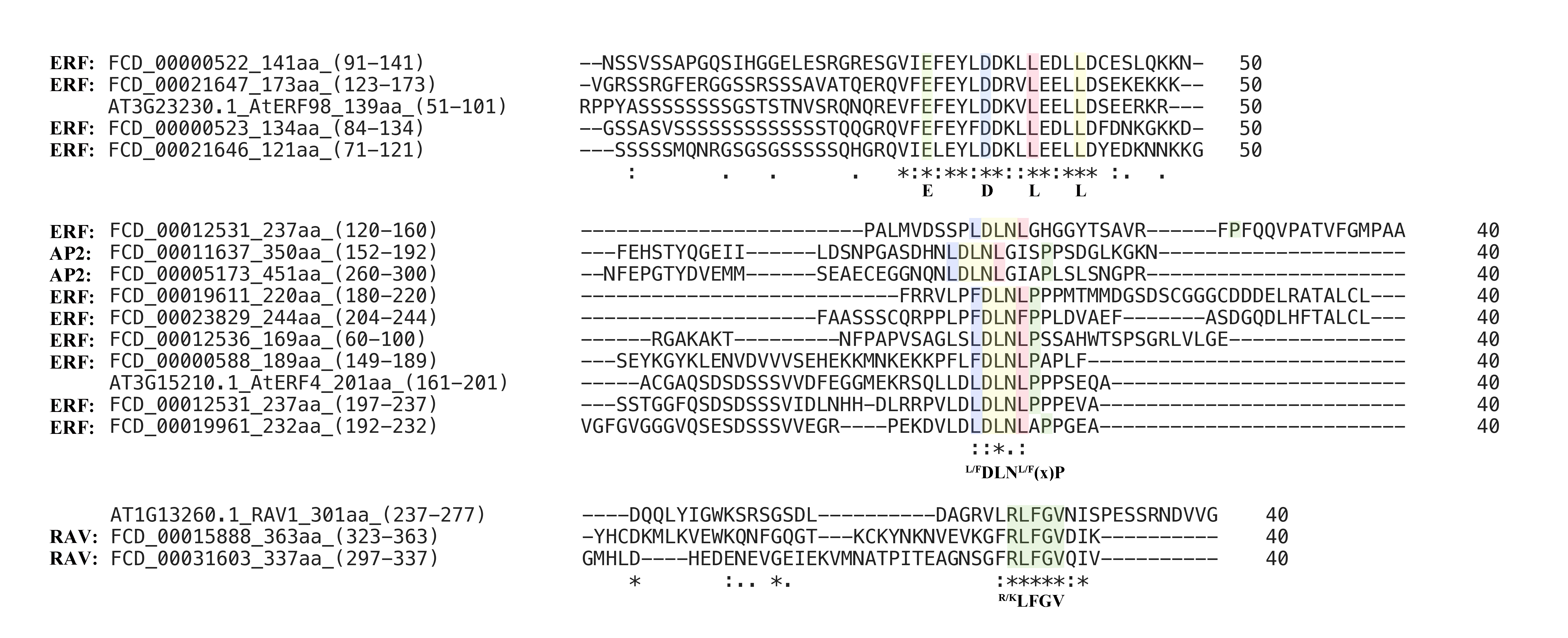

Supplement: Supplementary Figure 1 — EDLL activation domains, EAR repression domains, and R/KLFGV domains in FcAP2/ERFs. [file Image_1.jpeg]

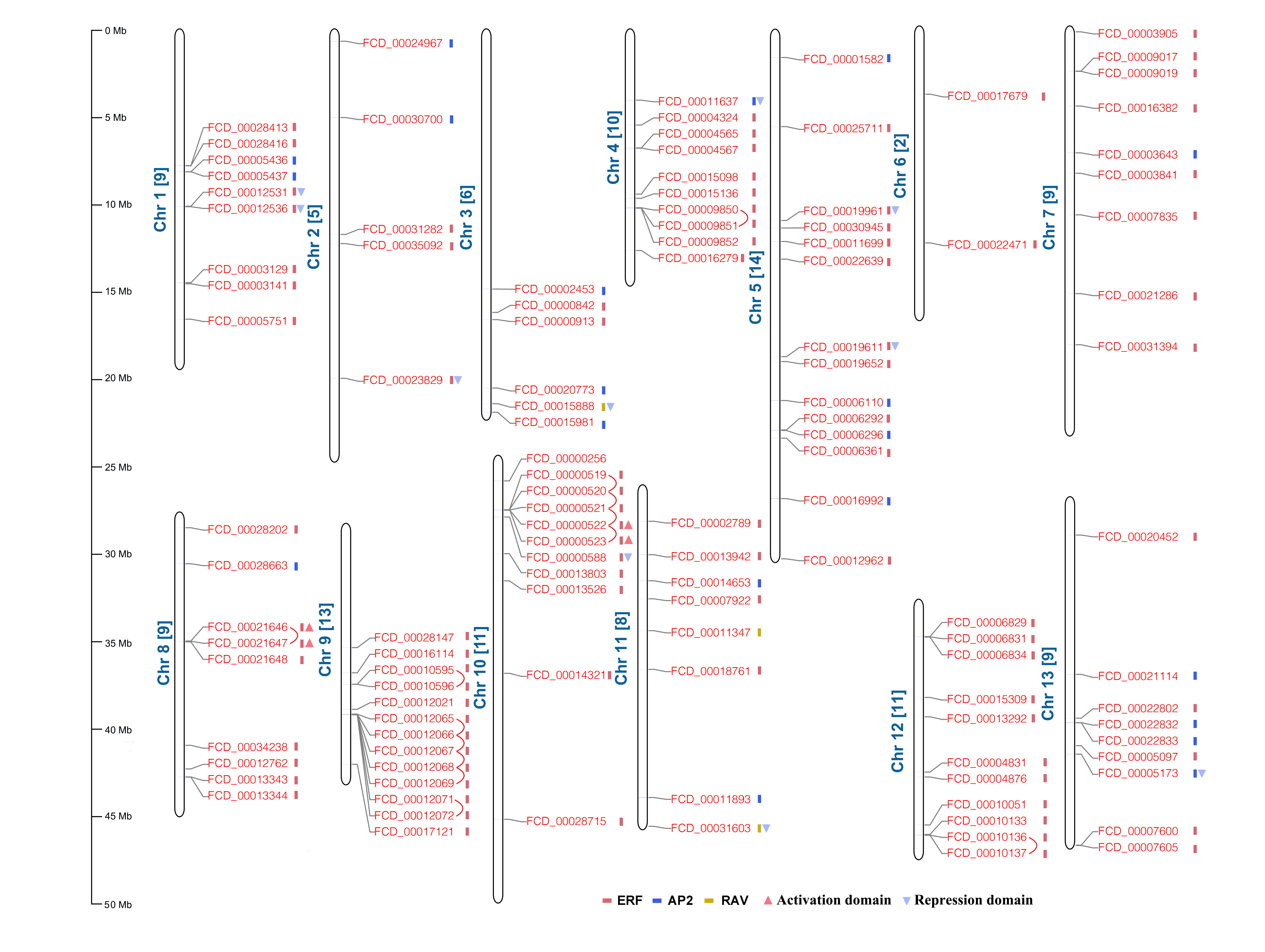

Supplement: Supplementary Figure 2 — Chromosomal distribution of FcAP2/ERFs. Red lines indicate AP2/ERF gene pairs derived from tandem duplication. The number after each chromosome name indicates the number of FcAP2/ERF genes mapped to that chromosome. [file Image_2.jpeg]

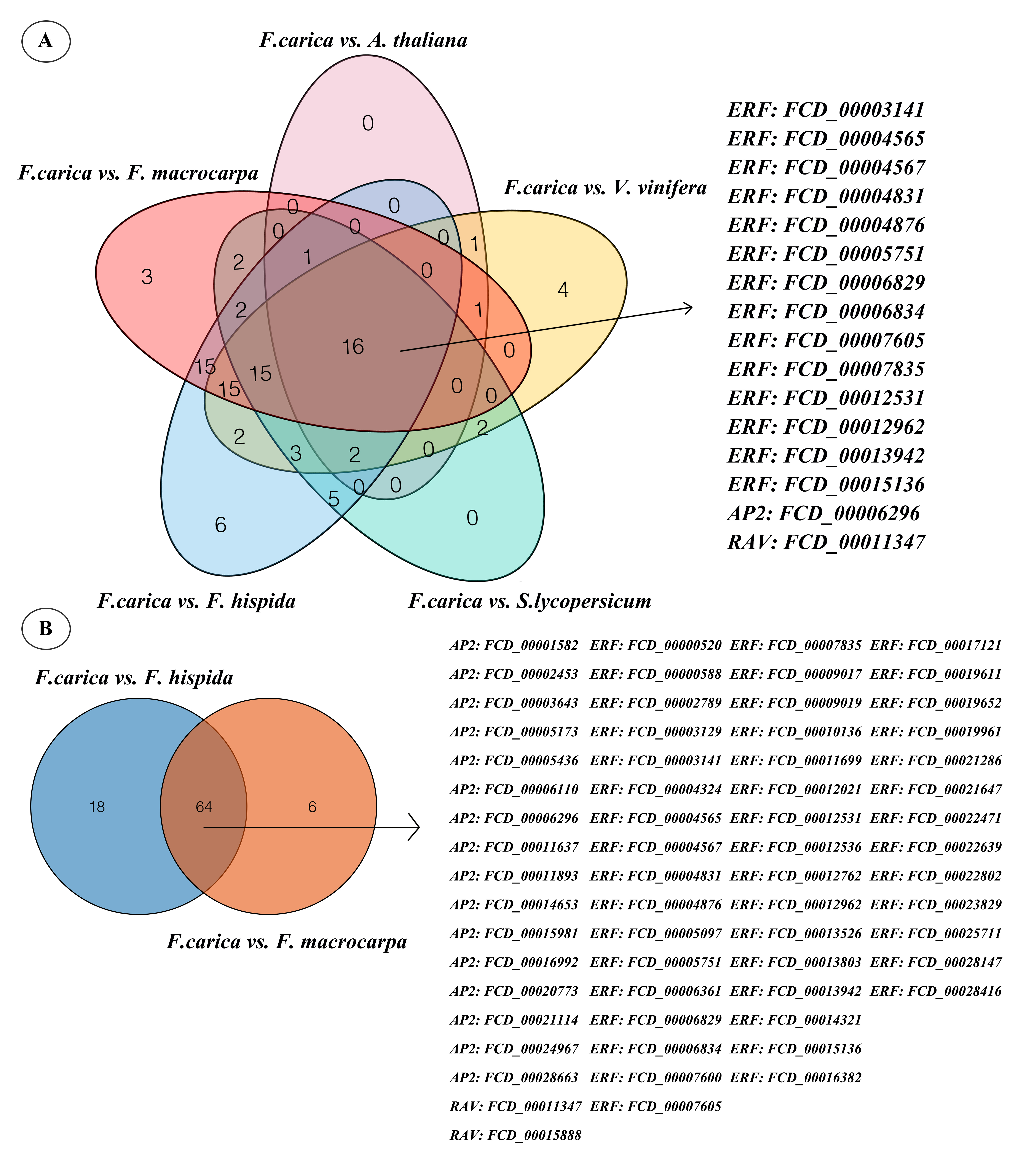

Supplement: Supplementary Figure 3 — Veen analysis of syntenic AP2/ERFs between F. carica and five other plant species. (A) FcAP2/ERF genes that showed syntenic relationships with all five plant species. (B) FcAP2/ERF genes that showed syntenic relationships with the other two Ficus species, F. hispida and F. macrocarpa. [file Image_3.jpeg]

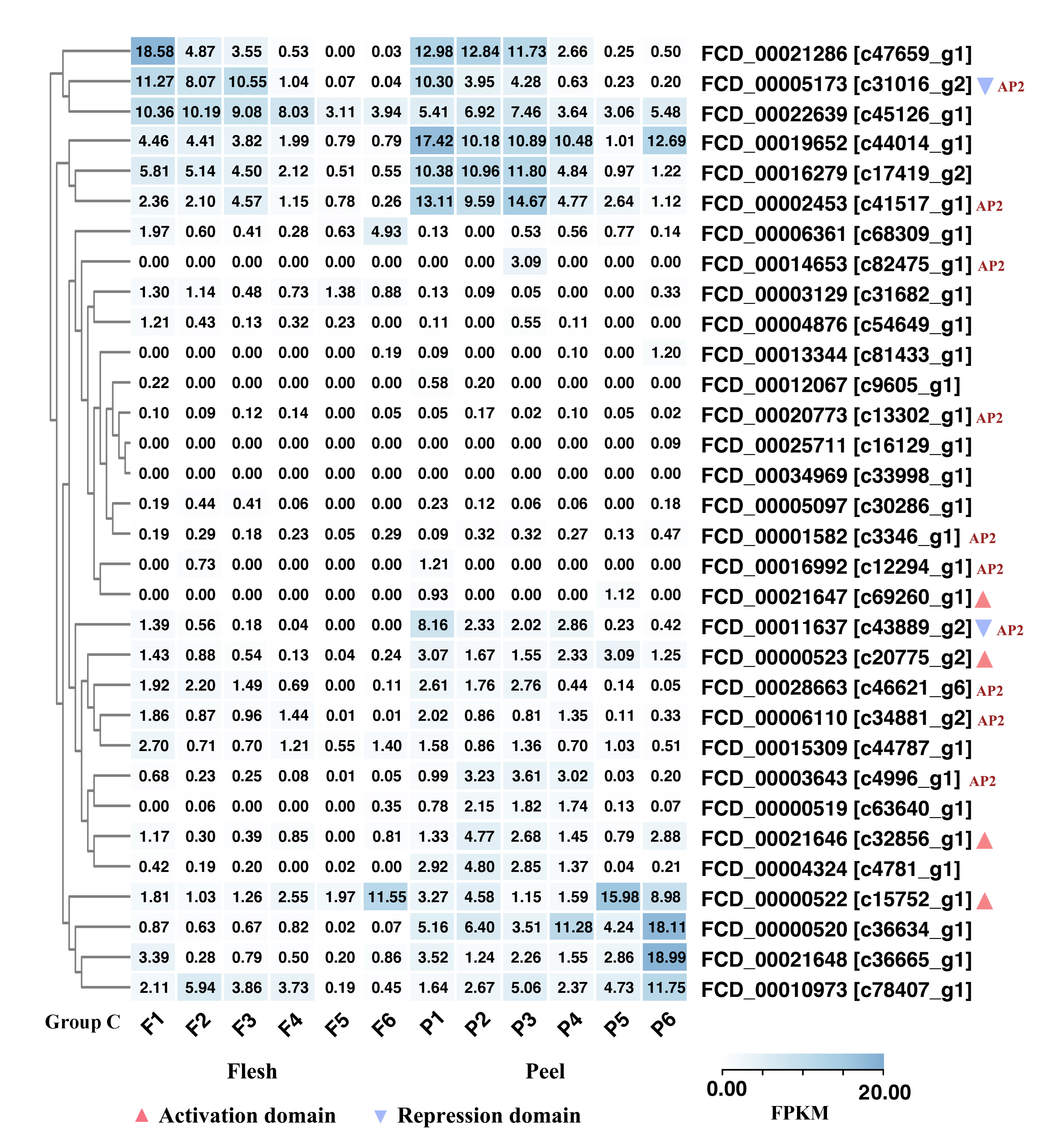

Supplement: Supplementary Figure 4 — Expression patterns of lowly-expressed (group C) genes at six fruit developmental stages. [file Image_4.jpeg]

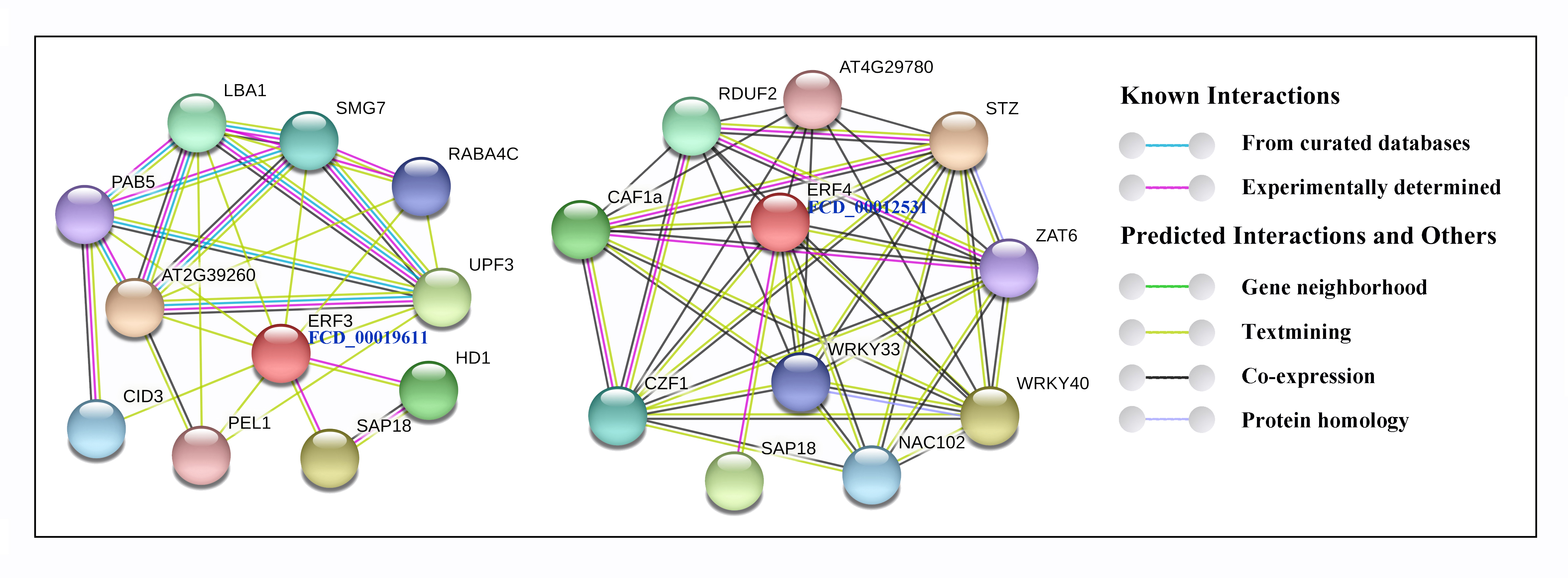

Supplement: Supplementary Figure 6 — Interaction networks of FCD_00019611 and FCD_00012531 based on those of A. thaliana homologs. [file Image_6.jpeg]

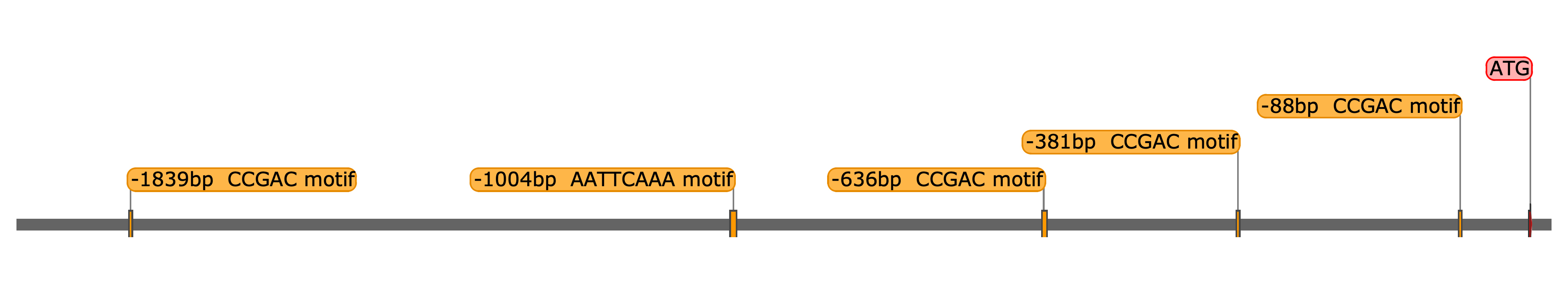

Supplement: Supplementary Figure 7 — AP2/ERF binding element in the promoter of pectate lyase. [file Image_7.jpeg]
